# Short and Long-Term Potential Role of Carbon Nanoparticles in Total Thyroidectomy with Central Lymph Node Dissection

**DOI:** 10.1038/s41598-018-30299-8

**Published:** 2018-08-09

**Authors:** Shuai Xue, Peiyou Ren, Peisong Wang, Guang Chen

**Affiliations:** Department of Thyroid Surgery, The 1st hospital of Jilin University, Changchun, 130021 People’s Republic of China

## Abstract

Whether we should use carbon nanoparticle (CN) routinely in thyroid surgery is still controversial. 406 papillary thyroid cancer (PTC) patients who underwent total thyroidectomy (TT) with bilateral central lymph node dissection (CLND) from January 2010 to December 2012 were retrospectively analyzed. The incidence of transient hypoparathyroidism and hypocalcemia in CN group was significantly lower than the control group at second, fifth day after surgery (*P* = 0.004, 0.042, 0.002 and 0.045 respectively). However, no significant difference existed between the two groups about the permanent hypoparathyroidism and hypocalcemia (*P* = *1.000*). Total number of central lymph nodes and metastatic lymph nodes in CN group were more than those in control group (*P* = 0.031 and 0.038 respectively). However, recurrence was not significantly different between the two groups after at least 5-year follow up (*P* = 0.7917). In the subgroup of prophylactic and therapeutic CLND study, no significant difference existed between the two groups (*P* = 0.5295 and 0.8459 respectively). CN significantly help in identifying the parathyroid glands in surgery and increased the number of lymph nodes in central compartment. However, we should not exaggerate the function of CN since it couldn’t improve the permanent hypoparathyroidism and recurrence in PTC patients who underwent TT with bilateral CLND.

## Introduction

Thyroid cancer is the most common endocrine malignancy. Among them, papillary thyroid carcinoma (PTC) is the most common subtype, accounting for more than 90% of all thyroid cancers. Usually, most patients with PTC obtain a 10-year survival rate of 80–90%, but the regional recurrence rate is up to 5–20%^1^. Central lymph node metastasis (CLNM) is proved to be an important factor influencing the tumor recurrence. As previously reported, the lymph node metastasis rate in PTC varies from 20% to 50%. Furthermore, nearly 50% of clinical-negative lymph node patients were diagnosed to be pathological-positive with micrometastases in lymph nodes^2–5^. Therefore, there is widely awareness that improving the central lymph node dissection (CLND) during operation can reduce the recurrence in PTC.

As a common and severe complication, the postoperative transient hypoparathyroidism incidence rate can be up to 37%^6,7^, which often caused by accidental resection of parathyroid glands or inadvertent injury to surrounding blood supply. Owing to negative impact on patients’ quality of life, it is particularly important to improve the identification of parathyroid glands and protect the blood supply during operation. Until now, no technique of distinguishing parathyroid glands such as methylene blue, parathyroid scintigraphy or frozen specimen biopsy has been considered as perfection.

Carbon nanoparticle (CN) is a novel lymph nodes tracer and has been widely used in the surgeries of breast cancer, stomach cancer and thyroid cancer^8^. During the surgery, the thyroid gland can be quickly black-stained after injecting the CN, but the anatomic color of the parathyroid will not change, which facilitates the surgeons to identify the parathyroid. Meanwhile, it can also stain the lymph nodes in the thyroid drainage area, which guides the surgeons to dissect the central lymph nodes.

In recent years, some studies suggested that CN could help to detect lymph nodes and increase the number of clinical apparent and microscopic metastatic lymph nodes^9–19^. Therefore, owing to its potential function of reflecting the metastatic condition and protecting parathyroid glands, the CN was recommended in thyroid surgery. However, Xu Liu *et al*. demonstrate CN is not beneficial for protecting the function of parathyroid gland in thyroid surgery from the perspective of declining intact parathyroid hormone (iPTH)^20^. Whether we should use CN routinely in thyroid surgery is still controversial. Moreover, the benefits of CN for recurrence of thyroid cancer are still unknown. Accordingly, we performed the retrospective study to clarify clinical benefits of CN in PTC patients with total thyroidectomy (TT) plus bilateral CLND.

## Materials and Methods

### Patients

406 patients with PTC were selected for this study. All selected patients underwent the total thyroidectomy (TT) with bilateral CLND (including prophylactic and therapeutic CLND). The operation was performed at the First Hospital of Jilin University from 2010 to 2012. The inclusion criteria were: (1) Patients underwent TT with bilateral CLND according to indications from America Thyroid Association (ATA) guideline in 2009; (2) Patients were diagnosed by fine needle aspiration (FNA) preoperatively; (3) Pathologically proven PTC; (4) Operations were completed by the same surgeon. The exclusion criteria were: (1) Reoperation; (2) Lateral lymph node metastasis (LLNM) and distant metastasis; (3) patients who have family history of thyroid cancer or head and neck were exposed to radiation; (4) non-PTC carcinomas (follicular/medullary/anaplastic); (5) Follow-up duration less than 6 months. During the study, CN was used on 106 patients. Meanwhile, 300 cases who did not use CN were selected as the control group. The study was approved by the First Hospital Ethics Committee of Jilin University. All of the experimental protocols and procedures were approved by the licensing committee and performed in accordance with the approved guidelines and regulations. The patients were informed that the clinical data would be used in the in this study, and informed consent was obtained from all of the subjects.

### CN injection

**Figure 1:**
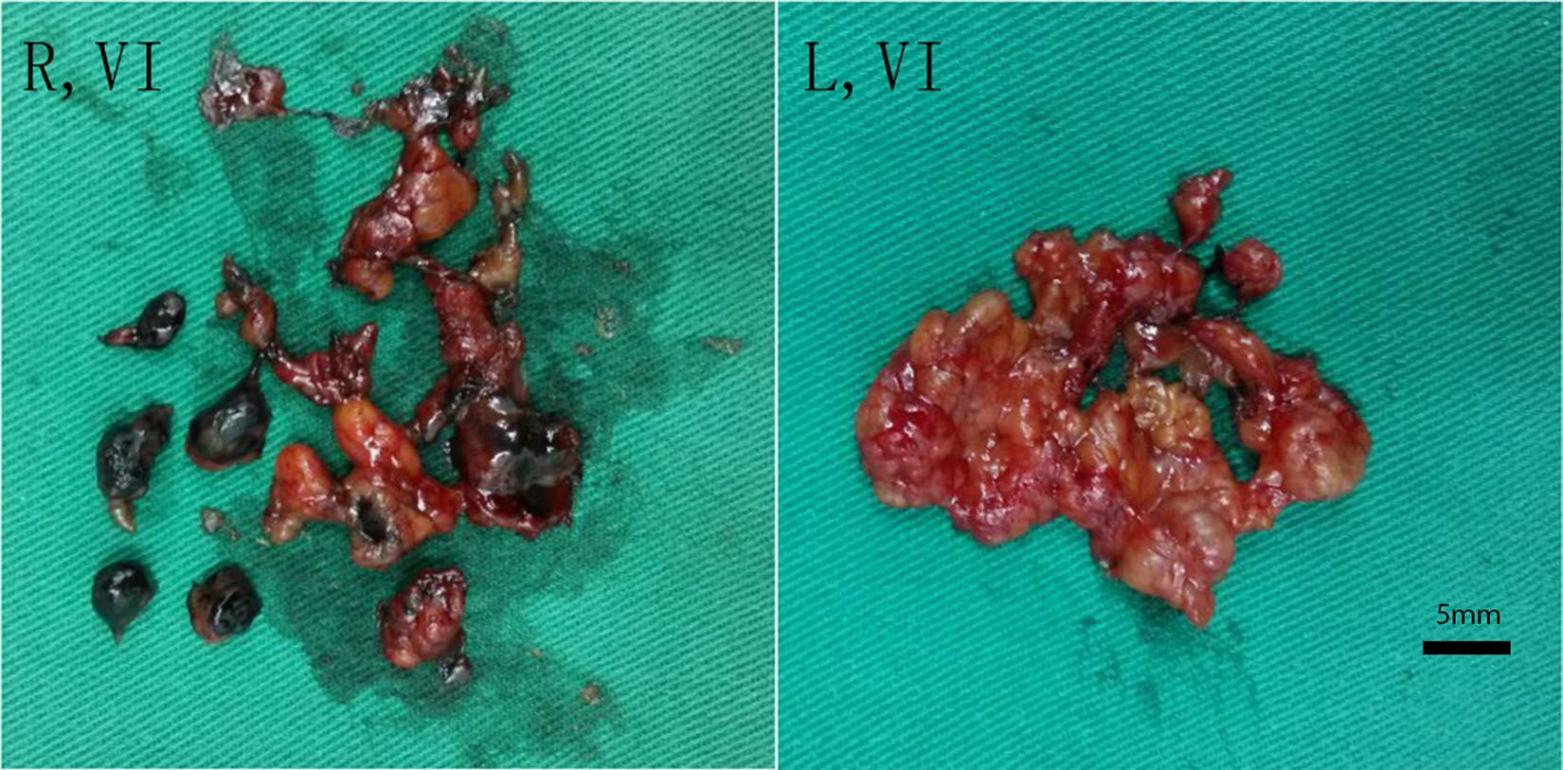
The number of central lymph node dissection. (R, VI), the dissected lymph nodes on the right thyroid lobe of the right central area in CN group; (L, VI), the dissected lymph nodes on the left side of the central area for the same patient without CN injection.

CN Suspension Injection (trade name: Kanalin) was designed by Chongqing LUMMY Pharmaceutical Co., Ltd. Specific processes (like organic solvent dissolution; magnetically stir, distillation and ultrasonic processing) changed aggregation of nanoscale carbon particles and formed polymer with 150 nm diameter. The gap between capillary lymphatic endothelial cells is 100–500 nm while that between the capillary endothelial cells is only 30–50 nm. Therefore, after being injected into the thyroid tissue, they will rapidly enter the lymphatic vessels, rather than vascular vessels, and then enter the lymphatic capillaries after having been engulfed by macrophages. Finally, they accumulated in the lymph nodes, resulting in the black-staining of the lymph nodes (Fig. 1). The CN suspension injection is mainly used for tracing the regional lymph node lesions. Usually after the exposure of the operative field, it can be injected at the amount of 25 mg with a 1 ml needle slowly and injected at the four to six points in the tumor periphery area. Each point of injection ranges from 5–15 mg and it takes about 3 minutes. Rare adverse effects are reported. Occasional fever occurred, which patients often could tolerate.

### Surgical procedures for the CN group

The type of CN Suspension Injection we used is 0.5 ml containing 25 mg with batch number: GUOYAOZHUNZI H20073246. After cutting the neck middle line, the thyroid was revealed. Before both sides of the thyroid gland being separated and the capsule being damaged, CN was injected using a 1 mL syringe. Two to five spots were injected at the lesion site. Depending on the size of the thyroid gland, total amount of CN injection is 8 to 20 mg. Both sides of the lobes are injected with CN. And before being injected, CN was preheated to 37 degrees. The syringe with CN was inserted deeply and aspirated to ensure the CN would not be injected into the blood streams. After injection, gauze was applied over the site to avoid drug leakage. After five to ten minutes, TT and CLND can be continued. This study included cases with both therapeutic and prophylactic CLND. Therapeutic CLND was performed when suspicious lymph node was detected during preoperative or intraoperative examination. CLND was performed to remove of all nodes and fibro-fatty tissue extending laterally from the medial border of the common carotid artery to the midline of the trache and vertically from the hyoid bone to the thoracic inlet. All the thyroidectomy specimen and lymph node tissues were sent for pathological examination.

### Date was recorded and evaluated for both groups

Both CN group and the control group were tested for the intact parathyroid hormone (iPTH), serum calcium level at second, fifth day, one month and six months after surgery. The number of lymph node was subject to the pathology postoperatively. Postoperative hypocalcemia and hypoparathyroidism, total number of central lymph nodes and metastatic lymph nodes were recorded and evaluated during the study.

### Postoperative treatment and follow-up

Patients with CLNM were receiving radioactive iodine ablation (RAI) if their tumors were larger than 1 cm and the capsule was invaded. All patients were receiving endocrine repressive therapy from 2011. A period of 60 to 84 months followed-up was conducted for all patients. Neck ultrasound, computed tomography (CT) scan, thyroglobulin (Tg) level and radioiodine whole body scan were used to diagnosis of recurrence and distant metastasis; When recurrence was suspected, patients underwent FNA with or without measurement of washout Tg levels and/or a thyroid CT or a positron emission tomography/CT. In our study, recurrence was defined as the presence of tumor or metastatic lymph node at least 6 months after the initial surgery. Permanent hypoparathyroidism was diagnosed as the iPTH and calcium level in the peripheral blood were still lower than normal after six months since surgery.

### Statistical analysis

SPSS version 22 software (SPSS Inc, Chicago, IL) was used for statistical analysis to identify differences between groups for specific variables. Data on categorical characteristics were expressed as absolute numbers. Continuous data were expressed as mean ± standard deviation (SD). The *x*^2^ test or Fisher’s exact test were used for categorical data, and t-tests or the Mann-Whitney test were used for continuous data. Survival curves were drawn by Kaplan-Meier method and statistically analyzed by the log-rank test. A p value < 0.05 was considered statistically significant.

### Data availability

The datasets generated during and/or analyzed during the current study are available from the corresponding author on reasonable request.

## Results

### Characteristics of patients

The clinicopathological characteristics of patients enrolled in the study are summarized in Table 1. Among the selected 406 patients, eighty-six cases were males and 320 were females, with a male-to-female ratio of 1: 3.72. The ages of patients ranges from 18–75 years with an average age of 44.88 years. There was no significant difference about patients’ age, gender, tumor location, multifocality, extrathyroidal extension (ETE), presence with thyroid nodule, presence with lymphocytic thyroiditis, tumor diameter, preoperative iPTH, preoperative calcium, RAI and follow-up period between the two groups (Table 1).

**Table 1:** Clinical characteristics of patients in the CN group and the control group.

	CN group (N = 106)	Control Group (N = 300)	*P value*
Age (year)	44.88 ± 7.78	44.35 ± 10.28	0.771
Gender			
Male	20	66	0.498
Female	86	234	
Tumor location			
Unilateral	16	72	0.056
Bilateral	90	228	
Multifocal tumors			
Unifocal	7	27	0.444
Multifocal	99	273	
ETE			
Yes	81	231	0.528
No	25	69	
With nodular goiter			
Yes	76	267	0.902
No	28	33	
With lymphocytic thyroiditis			
Yes	54	169	0.338
No	52	131	
Tumor diameter (cm)	1.56 ± 0.70	1.27 ± 0.80	0.056
Preoperative iPTH (pg/ml)	37.92 + 18.025	41.25 ± 18.01	0.347
Preoperative calcium			
(mmol/L)	2.23 ± 0.0897	2.20 ± 0.11	0.112
RAI			
Yes	55	168	0.464
No	51	132	
Follow-up period (months)			
Average ± SD	72 ± 9	71 ± 9	0.954
Outcome			
Tumor recurrence (%)	3/106	8/300	1
Disease-related death (%)	0/106	0/300	1

### iPTH and Calcium level after surgery

The incidence rate of hypoparathyroidism in CN group was significantly lower than that of the control group at day two, day five and one month after surgery (*P* = 0.002,0.045 and 0.033 respectively). However, a six-month follow-up showed no significant difference between the two groups about the postoperatively permanent hypoparathyroidism (*P* = 1.000) (Table 2).

**Table 2:** Number of hypocalcemia and hypoparathyroidism postoperative in CN group and control group.

Test items	Post time	CN Group (N = 106)	Control Group (N = 300)	*P value*
Hypoparathyroidism (<8 ng/ml)	2 days	8 (7.5%)	63 (21%)	0.002
	5 days	7 (6.6%)	42 (14%)	0.045
	1 month	2 (1.9%)	23 (7.7%)	0.033
	6 months	1 (0.9%)	3 (1%)	1
Hypocalcemia (<1.9 mmol/L)	2 days	10 (9.4%)	66 (22%)	0.004
	5 days	8 (7.5%)	46 (15.3%)	0.042
	1 month	4 (3.8%)	19 (6.3%)	0.327
	6 months	1 (0.9%)	3 (1%)	1

The number of patients with hypocalcemia in CN group was significantly less than the control group at day two, day five after surgery (*P* = 0.004 and 0.042 respectively). However, the long-term follow-up found no significant difference of the incidence of permanent hypocalcemia (*P* = 0.327 and 1.000 respectively) (Table 2).

### CLND

**Figure 2:**
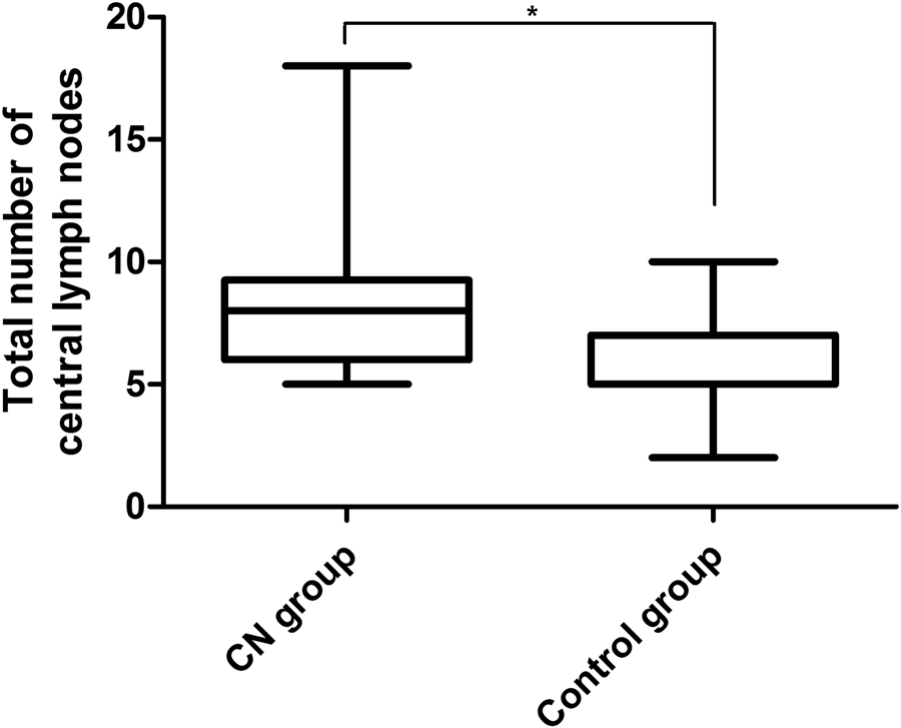
The total of the central lymph node dissection, both the upper four and median number in the CN group were higher than the control group (*P* = 0.031).

**Figure 3:**
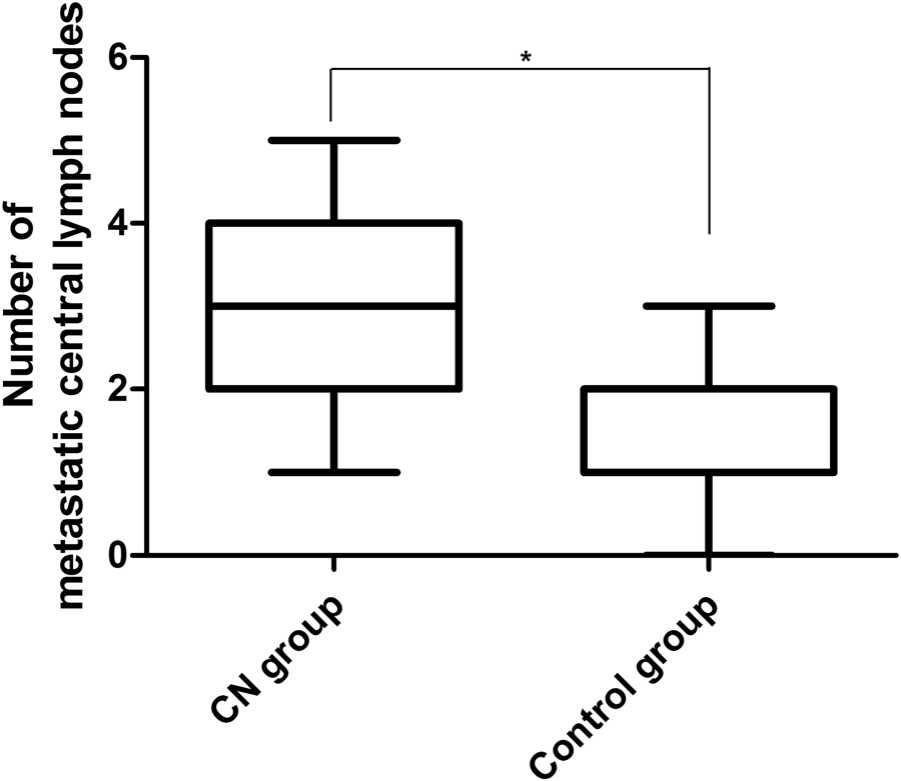
The total number of central positive lymph nodes dissection (including the positive lymph nodes is “zero”), the upper four numbers in the CN group were higher than the control group (*P* = 0.038).

Total number of central lymph nodes and metastatic lymph nodes in CN group were more than those in control group (*P* = 0.031 and 0.038 respectively; Table 3; Figs 2 and 3). The lymph node metastatic rate was 59.44% and 52% respectively in the CN group and the control group (*P* = 0.187; Table 4).

**Table 3:** Number of central lymph node dissection in CN group and control group.

Test items	CN Group M (P25, P75)	Control Group M (P25, P75)	*P value*
The total number of central lymph nodes	8.0 (4.0, 13)	5.0 (3.0, 7.0)	0.031
The number of metastatic lymph nodes	3.0 (0.0, 5.0)	1.0 (0,0,2.3)	0.038

Note: M (P25, P75) = Median, Lower Quartile, Upper Quartile.

**Table 4:** Patients with CLNM.

Metastasis	CN group	Control group	*P value*
Yes	63 (59.4%)	156 (52%)	
No	43 (40.6%)	144 (48%)	0.187

### Recurrence in the two groups

**Figure 4:**
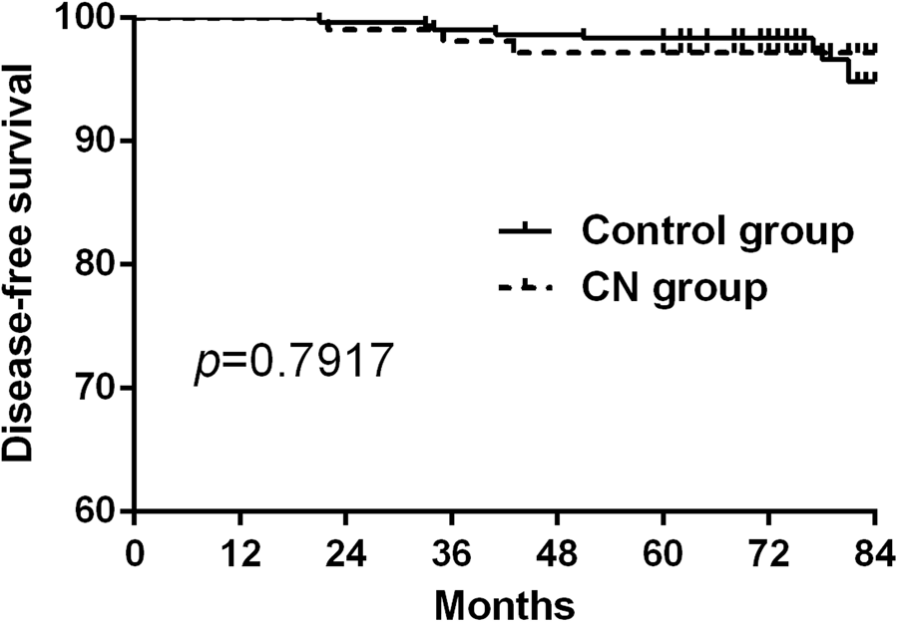
There was no significant difference of the disease-free survival between the CN and control group. (*P* = 0.7917).

**Figure 5:**
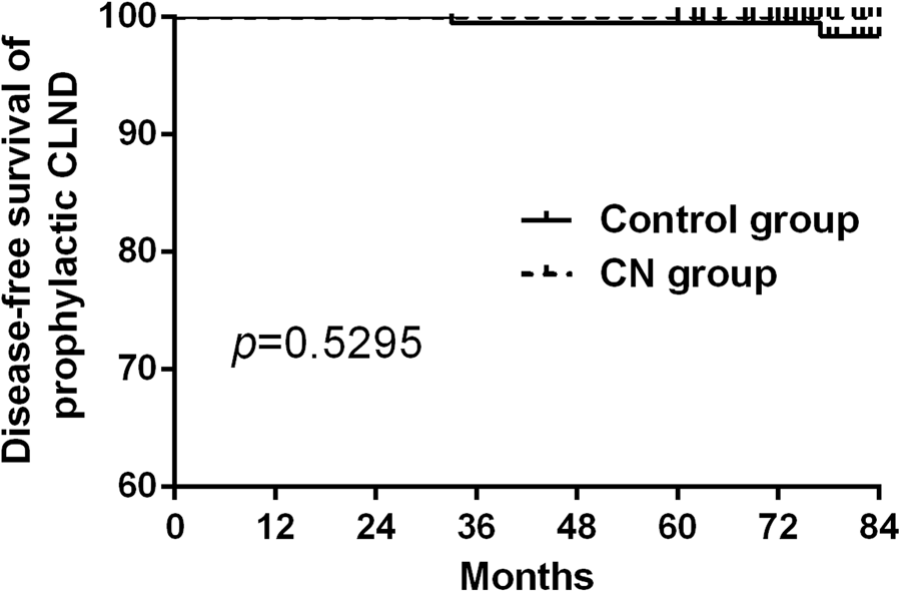
There was no significant difference of the disease-free survival for prophylactic CLND between the CN and control group (*P* = 0.5295).

**Figure 6:**
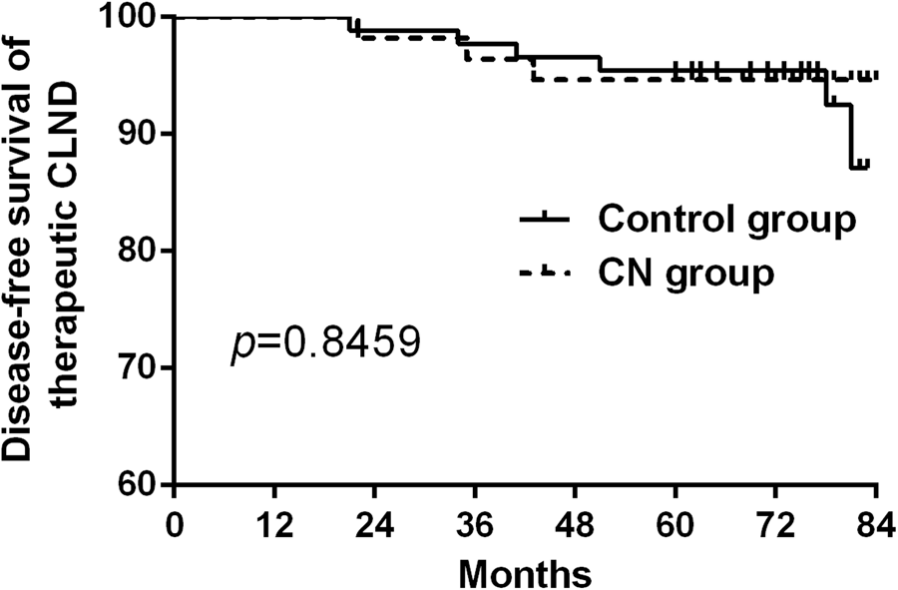
There was no significant difference of the disease-free survival for therapeutic CLND between the CN and control group (*P* = 0.8459).

All patients were followed up from 60 to 84 months, there were 3 recurrent cases in the CN group and 8 cases in the control group. Disease-free survival curve was drawn by Kaplan-Meier method and statistically analyzed by the log-rank test to find that there was no significant difference between the two groups (*P* = 0.7917, Fig. 4). In the subgroup of prophylactic and therapeutic CLND study, we also found that no difference existed between the CN group and control group (*P* = 0.5295 and 0.8459 respectively, Figs 5 and 6).

## Discussion

Total or near-total thyroidectomy plus central lymph node dissection was usually underwent for PTC. Parathyroid injury is the most common complication of TT which could induce hypoparathyroidism^8^. As studies reported, parathyroid injury rate has been reduced to some extent due to the awareness of the parathyroid protection and the operative skill. However, the postoperative parathyroid injury rate is still up to 0.3–49%^6,7^. In addition, the parathyroid blood was supplied by superior and inferior thyroid arteries, and the blood supply for parathyroid would be impaired by TT. Injury to parathyroid would lead to hypocalcemia, which is often caused by accidentally removing or destroying of the patients’ parathyroid glands or inadvertent destroying the glandular blood supply. Hypocalcemia could lead to many neuromuscular symptoms, such as sense of numbness or stiffness around face, mouth or foot; Serious symptoms would be muscle spasms and pain on hand, foot, or even suffocation caused by the serious spasm on throat and diaphragm. Therefore, protecting parathyroid and keeping its blood supply intact are always a main topic for TT^21^.

Recently, CN suspension injection has been used as an excellent tracer for lymph nodes in the gastric and gynecological tumors. Through the effect of the CN suspension, the surgeon would be able to identify the parathyroid, which can improve the protection of the parathyroid. In this study, we found that the incidence rate of hypoparathyroidism and hypocalcemia in CN group was significantly lower than that of the control group in a short period (P < 0.05). However, long-term follow-ups had found no significant difference between the two groups in the incidence of permanent hypoparathyroidism and hypocalcemia. The result of the CN suspension application in the thyroid surgery was consistent with the research result reported by Wang Bin *et al*.^22^. The plausible explanation is early detection of parathyroid using CN help surgeons to protect blood vessels of parathyroid, which leads to decreased rate of hypoparathyroidism and hypocalcemia at short-term follow up. At long-term (6 months), reconstruction of blood vessels for parathyroid can rescue its impaired function during surgery without CN. From the point of improving the iPTH and serum calcium level, the CN suspension has an important clinical value in protecting of parathyroid glands in the TT.

For PTC, neck regional recurrence rate after surgery is 5–20% and CLNM is an important source of recurrence^2–5^. Therefore, improving the thoroughness of neck lymph node dissection can reduce the rate of tumor recurrence^4,5^. Recurrence rate in high risk group was significantly higher than the low risk group (14% vs 34%), and the recurrence rate in lymph node metastasis is higher than those patients without metastasis^23^. Prophylactic CLND is still controversial in recent years. Studies have shown that there is no difference in the recurrence rate between the suspicious cervical lymph node metastasis in patients with unilateral CLND or bilateral CLND^24–26^. Some studies have also found that there is no difference in recurrence rate between the TT and TT + bilateral CLND in PTC patients, but the number of transient hypoparathyroidism was significantly increased in the CLND group^27^. However, prophylactic CLND is also traditional procedure for median to high-risk group of patients. Therefore, it is particularly important to dissect central lymph nodes thoroughly and reduce the hypoparathyroidism.

Consistent with the literature reported, the total number of lymph nodes and metastatic lymph nodes for TT in CN group was significantly more than those in the control group^9–19^. The difference was statistically significant, which indicated that CN can improve CLND without increasing the injury rate of parathyroid. As Table 3 shown in our study, CN can increase the total number and metastatic central lymph node at the same time. The total central lymph node metastasis rate was 54.25%, while the CN group was 59.44% and the control group was 49.01%. CN group is slightly higher, but the difference was not statistically significant, which indicated that CN suspension injection can improve the thoroughness of CLND. Moreover, by long-term follow up there was no significant difference between the two groups in disease-free survival. In the subgroup of prophylactic and therapeutic CLND study, we also found that no difference existed between the CN group and control group.

So far, few side effects related to CN suspension injection had been reported. During the surgery, drug staining in the exterior of thyroid tissue can affect observation in the surgical field and increase the difficulty to identify the laryngeal nerve. It can also stain the skin if medication was not carefully administered during surgery. But the risk could be avoided if the operation standards were strictly followed.

Our study is retrospective, which means the evidence we provided may not as powerful as a multi-centric pragmatic randomized controlled clinical trial with large population. Moreover, TSH suppressive hormonal therapy was applied to postoperative patients after 2011. Because at that time, there was no guideline which we could follow to recommend TSH-suppressive hormonal in China. However, to our knowledge, this is the first article in English to evaluate the long-term outcomes of PTC patients who using CN during operation, which provided significant information surgeons could used for clinical recommendation.

## Conclusion

In this study, we demonstrated that the use of CN significantly helped in identifying the parathyroid glands in surgery, which improved the postoperative iPTH and serum calcium level and decreased the incidence of transient hypoparathyroidism. Moreover, it also notably increased the number of total and metastatic lymph nodes in central compartment. However, we should not exaggerate the function of CN since it couldn’t improve the permanent hypoparathyroidism and recurrence in PTC patients who underwent total thyroidectomy with bilateral central lymph node dissection.
